# Salient Object Detection with Semantic-Aware Edge Refinement and Edge-Guided Cross-Attention Feature Aggregation

**DOI:** 10.3390/s26082439

**Published:** 2026-04-16

**Authors:** Yitong Lu, Ziguan Cui

**Affiliations:** 1 Portland Institute, Nanjing University of Posts and Telecommunications, Nanjing 210023, China; p23000617@njupt.edu.cn; 2 College of Telecommunications and Information Engineering, Nanjing University of Posts and Telecommunications, Nanjing 210003, China

**Keywords:** salient object detection, dual-encoder saliency network, semantic-aware, edge refinement, cross-attention

## Abstract

Hybrid multi-backbone architectures and the utilization of edge cues for auxiliary training have become two major research trends in salient object detection (SOD). It is widely acknowledged that CNNs can effectively model local spatial structures, while Transformers can capture long-range global dependencies. However, the representation discrepancy between CNN and Transformer features, together with boundary-detail degradation during multi-scale fusion, remains a major challenge. In addition, how to effectively leverage edge cues as reliable structural guidance without introducing texture-induced false boundaries or boundary leakages remains an open issue. In this paper, we present SECA-Net, a unified framework that establishes a profound synergy between CNN and Transformer representations. It explicitly bridges their inherent discrepancies through level-dependent interaction strategies, while resolving structural degradation via a sequential “purify-and-guide” mechanism. This approach enables the network to extract and utilize edge cues effectively, thereby alleviating boundary degradation and texture-induced false contours. Specifically, we design a dual-encoder structure to extract features. A level-wise feature interaction (LFI) module is introduced to perform discrepancy-aware fusion across feature levels, stabilizing CNN–Transformer aggregation. Meanwhile, the features extracted from the CNN branch are projected into a semantic-aware edge refinement (SAER) module to produce clean multi-scale edge priors under high-level semantic guidance, suppressing texture-induced spurious edges. Finally, we design an edge-guided cross-attention feature aggregation (ECFA) module, which progressively injects refined edge priors as structural constraints into multi-scale saliency decoding via cascaded cross-attention, enabling effective structural refinement. Overall, LFI reduces cross-branch discrepancy, SAER purifies boundary priors, and ECFA integrates semantics and structure in a progressive decoding manner, forming a unified SECA-Net framework. Extensive experimental results on five benchmark SOD datasets show that SECA-Net outperforms 19 state-of-the-art methods, demonstrating its effectiveness. Specifically, our proposed method ranks first in $F_{\beta }$ and BDE across all datasets, notably improving $F_{\beta }$ by 1.54% on the challenging DUTS-TE dataset.

## 1. Introduction

Salient object detection (SOD), which simulates the human visual perception mechanism, aims to identify the most distinctive regions in natural scenes and has been extensively used in a range of downstream vision tasks, such as scene classification, image captioning and semantic segmentation. Traditional approaches, which rely on handcrafted priors or low-level cues, often fail in complex backgrounds and lack the ability to capture high-level semantics.

With the development of deep learning [1,2,3], CNN-based models [4,5,6] have demonstrated their impressive capabilities in extracting local detailed features. However, the local receptive fields restrict the capabilities of CNN-based models [7] to capture sufficient global context information. This limitation often leads to failures on images with complicated structures and strong background clutter. In recent years, Vision Transformers (ViT) [8], equipped with stacked self-attention layers, have shown strong capabilities in capturing better long-range dependencies and precise localization. Subsequently, Transformer-based models [9] have been proposed for SOD.

As is well known, CNNs and Transformers demonstrate strong complementarity. Transformers capture long-range dependencies in global modeling, enabling sufficient global context representation. CNNs, with their local receptive fields, are better at preserving spatial structural details. How to achieve a more efficient integration of the two representations has emerged as a major challenge in current SOD research. Many hybrid designs rely on basic fusion schemes, such as addition, multiplication and simple concatenation, which may not adequately resolve the representation discrepancy. As a result, straightforward fusion can suppress discriminative details and lead to unstable integration across levels. To some extent, more complex convolution operations can alleviate this issue, but they hardly reconcile performance with computational efficiency. Furthermore, low-level features preserve fine-grained boundary and texture details but are sensitive to background clutter, whereas high-level features encode low-resolution yet robust object semantics, suggesting that cross-branch interaction should be designed in a level-dependent manner.

How to efficiently extract and make full use of edge information for improving boundary quality without degrading region-level saliency consistency is the second challenge. Some algorithms [10] excavate edge cues directly from saliency features under the supervision of edge labels and fuse them with saliency features by basic pixel-wise addition or multiplication. For example, DSRNet [11] uses morphological erosion and dilation operations on saliency features to acquire edge information. Recently, Wu et al. [12] investigated weakly supervised SOD and successfully suppressed pseudo-label noise by emphasizing two critical principles: spatial continuity and nonequal importance. Although existing methods improve performance, they often overlook the necessity of these principles. Specifically, interior features of salient regions must preserve strict spatial continuity to maintain completeness. Meanwhile, structural cues demand nonequal importance, meaning genuine object boundaries should be highly prioritized, whereas task-irrelevant texture noise must be heavily suppressed. As a result, treating all raw edge responses with equal attention may introduce texture-induced false boundaries, disrupt spatial continuity, and cause boundary leakage.

**Figure 1:**
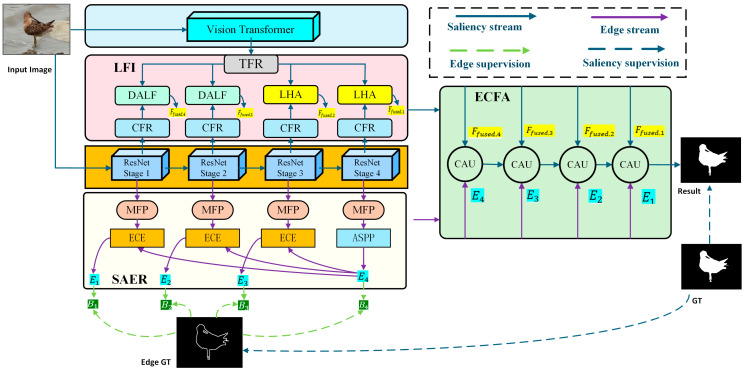
The architecture of the proposed SECA-Net.

To address these issues, we design a novel CNN-Transformer dual-encoder network with semantic-aware edge refinement and edge-guided cross-attention feature aggregation (SECA-Net) for SOD tasks, as illustrated in Figure 1. SECA-Net is a unified framework that comprises three tightly coupled significant components: the level-wise feature interaction (LFI) module, the semantic-aware edge refinement (SAER) module, and the edge-guided cross-attention feature aggregation (ECFA) module. Our method aims to provide a semantic–structure co-regularization principle, which reduces cross-branch discrepancy between CNN and Transformer features and injects refined structural priors into progressive decoding for boundary-sensitive saliency prediction. To be specific, we adopt parallel CNN and Transformer encoders to independently extract two groups of features. LFI serves as the interaction bridge between the two encoders, which performs level-wise fusion with dedicated submodules for lower-level and high-level features to alleviate cross-branch representation discrepancy. To tackle the limitations of current edge-guided models, we further propose a SAER, which refines multi-scale edge representations under object-aware semantic priors, suppressing task-irrelevant texture edges while preserving salient contours. In this way, SAER produces clean and reliable boundary priors that can be safely used to guide saliency decoding. Fundamentally distinct from existing edge-guided methods that treat edge supervision merely as a passive auxiliary task, the proposed SECA-Net introduces an active “purify-and-guide“ strategy. While conventional models often directly fuse raw edge cues into saliency features, which inevitably introduces texture-induced noise, our framework leverages SAER to actively purify structural priors under high-level semantic guidance. Furthermore, the ECFA module utilizes these refined edges as dynamic structure-aware signals rather than simple concatenated features. This bidirectional collaboration intrinsically prevents boundary leakage and texture interference. LFI reduces cross-branch discrepancy, SAER purifies structural priors, and ECFA integrates semantics and structure progressively, forming a unified framework. In general, our main contributions are as follows:

1. We propose SECA-Net, a novel CNN-Transformer dual-encoder framework for SOD. Instead of the traditional strategy of passive edge supervision, SECA-Net introduces an active “purify-and-guide” mechanism, ensuring that edge cues serve as reliable, structure-aware constraints rather than introducing texture-induced noise.
2. We design LFI to explicitly address the level-dependent heterogeneity between CNN and Transformer representations. Specifically, low levels use dynamic attention to calibrate cross-branch correspondence for low-level details and boundaries, while high levels perform semantic gating and residual enhancement for stable high-level fusion.
3. We propose SAER, which provides clean and reliable edge priors. SAER performs soft semantic selection with the top-level semantic guidance and further complements it with Sobel-derived local gradients, thereby suppressing task-irrelevant texture boundaries while preserving object-aware contours.
4. We design ECFA that treats the purified edges as dynamic structural signals within a top-down cross-attention framework, enabling bidirectional collaboration between interior semantics and boundary structures. Extensive ablations validate that each component is non-redundant and significantly contributes to boundary precision and structural consistency.

## 2. Related Work

SOD aims to identify the most visually prominent regions in an image and has demonstrated outstanding performance in a wide range of applications [13,14]. Up to now, numerous SOD networks have been designed. They can be divided into two main categories: traditional methods and deep learning methods. Traditional methods [15] mainly depend on handcrafted features such as contrast [16], boundary background, and so on. In contrast, deep learning methods [17] demonstrate superior performance to traditional methods.

### 2.1. Vision Transformer for SOD

The Transformer architecture was first proposed for natural language processing [18] in 2017. With the proposal of Vision Transformer (ViT) [8], Transformer-based methods for SOD have been inspired.

Unlike CNNs that primarily model local structures, Transformers capture long-range dependencies through multi-head self-attention, enabling stronger global reasoning and better contextual understanding. Swin Transformer variants further improve the efficiency and multi-scale representation capability of Transformer encoders, making them suitable for dense prediction tasks including SOD.

Recently, hybrid CNN-Transformer architectures have been explored to integrate complementary advantages from the two paradigms. For example, Yuan et al. [19] proposed CTIF-Net to combine CNN backbones with a Transformer encoder to jointly leverage global–local cues. It adopts iterative fusion, adding and concatenating the features of different levels together repeatedly to achieve information complementarity, which tends to ignore the level-dependent representation discrepancy. Li et al. [20] established hierarchical space-channel correlations within a single feature stream to derive discriminant representations. However, it could not leverage the native complementary advantages of parallel dual-backbone systems, which caused a deficiency in the ability to capture details. In fact, low-level features focus on rich pixel-level details such as edges and textures. The local receptive fields of CNNs and the global token relationships of Transformers exhibit intrinsic conflicts, which require precise spatial calibration. In contrast, high-level features are characterized by low resolution and high semantic abstraction. Naive fusion often leads to feature distortion and semantic redundancy. Motivated by the limitations, we propose LFI. At low levels, we design DALF to adaptively calibrate the importance of different channels for every individual pixel through dynamic channel-wise weighting at each spatial position, thereby resolving local representation discrepancies between CNN and Transformer branches. At high levels, LHA employs a multiplicative gating mechanism to activate consistent high-level responses while suppressing task-irrelevant features.

### 2.2. Edge-Guided Methods

Edge-guided learning excels at preserving structural integrity and improving boundary accuracy and has been widely adopted in SOD [21,22]. It collaborates with the interior of the salient features and enables a clear object contour.

Edge-guided streams are designed to directly learn edge details, collaborating with the saliency branch. For example, ELSA-Net [23] leverages edge-guided learning to guide the fusion of saliency information. As the edge region forms a subset of the saliency map, edge-guided loss functions are also used to guide the learning of edge details [24]. Recently, Yao et al. [25] introduced a multi-oriented edge enhancement (OEE) module to capture structural features from multiple directions by integrating square, strip, and diagonal kernels. However, they face two primary limitations. First, in ELSA-Net, the edge features are prematurely assisted in fusion without any processing. Consequently, any noise in the raw edge cues is immediately mixed into the saliency features, violating the principle of nonequal importance and potentially misleading the network in complex backgrounds. Second, RECE-Net couples edge cues and region saliency features together, lacking a mechanism to independently handle the spatial precision for complex object localization, which is crucial for maintaining spatial continuity.

To address these limitations, fundamentally distinct from methods that treat edge supervision merely as a passive auxiliary enhancement, our framework introduces an active “purify-and-guide” strategy. Specifically, we propose the SAER module, which serves as an independent geometric monitor. Our edge information is rooted in the CNN branch mapping, while isolating the connection with Transformer features, ensuring the purity of global information. We first designed the multi-scale feature preprocessing (MFP) module to extract initial structural cues. Crucially, a high-level structural prior is derived from the deepest stage and then back-propagated to calibrate lower-level features via a dual-gate mechanism, effectively enforcing nonequal importance by suppressing texture noise. This decoupled calibration strategy ensures that refined structural anchors effectively constrain the Transformer’s semantics within precise boundaries post-fusion, thereby guaranteeing the strict spatial continuity of the salient objects.

### 2.3. Attention Mechanism

Attention mechanisms, which are inspired by the human visual perception process, have played a vital role in modern salient object detection [26]. Early works primarily adopt spatial or channel attention to enhance CNN features. For example, PiCANet [27] introduces pixel-wise contextual attention to selectively aggregate information. With the emergence of self-attention, Transformer-based SOD models have been proposed to leverage multi-head self-attention to capture long-range dependencies across the entire image.

To achieve interaction across heterogeneous feature spaces, some recent approaches start to explore cross-attention mechanisms. For example, Zhu et al. [28] restrict cross-attention to the predicted masked regions to perceive image features, enabling the network to prioritize the overall area of salient objects. While it effectively localizes the body of salient objects, it often lacks sufficient constraints at the boundaries, which causes an inherent limitation. Relying solely on coarse masks for attention guidance frequently results in blurred object contours, as the network prioritizes interior regional consistency over precise structural alignment.

In this paper, we propose ECFA to overcome this region-boundary imbalance, where refined edge cues act as structure-aware guidance to modulate interior semantic features progressively. This design improves boundary localization and produces coherent saliency maps through semantic–structure collaboration.

## 3. Proposed Method

In this section, we elaborate on the overall architecture of the proposed SECA-Net. The remainder of this section introduces the three main components of our architecture: the LFI, the SAER, and the ECFA. Ultimately, we detail the loss functions.

### 3.1. Overall Architecture

The overall framework of our proposed method has been illustrated in Figure 1. Our encoder adopts a dual-branch design: a Transformer branch and a CNN branch. In the Transformer branch, the input image is first partitioned into non-overlapping 16×16 patches, as is customary in ViT-style models. After linear projection and positional encoding, the resulting tokens enter stacked Transformer blocks to produce high-level semantic representations. The CNN branch adopts ResNet-50, extracting four-level multi-scale features $F_{i\;}\left\{i=1,2,3,4\right\}$ from its hierarchical residual blocks. The outputs of the two branches are then fed into LFI for cross-branch feature interaction. After the interaction stage, we obtain four scales of fused CNN–Transformer features, denoted as $F_{i}^{fuse}\left\{i=1,2,3,4\right\}$. Meanwhile, the CNN features are projected into the SAER, which derives a set of edge features $E_{i}\;\left\{i=1,2,3,4\right\}$. Finally, the edge features $E_{i}$ and fused features $F_{i}^{fuse}$ are processed by ECFA, which performs top-down decoding to generate the final saliency map.

### 3.2. Level-Wise Feature Interaction Module

**Figure 2:**
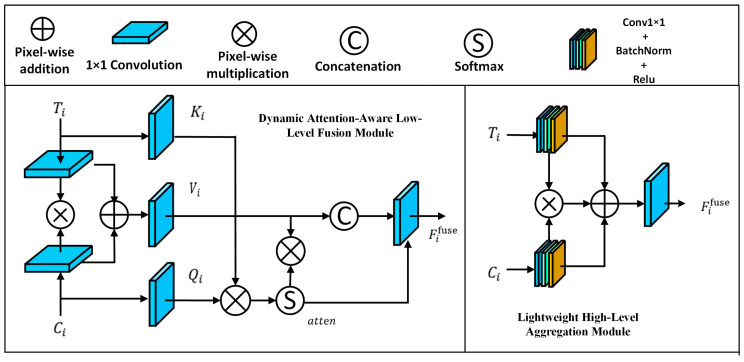
Structure of the level-wise feature interaction module. The LFI consists of a dynamic attention-aware low-level fusion model and a lightweight high-level aggregation module.

As is well known, CNNs work effectively at extracting rich spatial features, while Transformers excel in capturing global contextual information. Effectively integrating the complementary strengths of these two representations remains a crucial challenge in SOD. To address this issue, we design two interaction modules for features at different levels: the dynamic attention-aware low-level fusion (DALF) module and the lightweight high-level aggregation (LHA) module. Before feature interaction, we introduce a lightweight Transformer feature reformatter (TFR). This step converts each stage output and is simply compressed to the target channel dimension using a CNN feature reformatter (CFR) with a 1×1 convolution, without introducing any additional processing. Thus, we obtain two sets of four-level matching features: $T_{i},C_{i}\;\left\{i=1,2,3,4\right\}$, which are shown in Figure 2.

(1) Dynamic attention-aware low-level fusion module

Low-level features are characterized by high spatial resolution and rich local details. The shallow features of CNNs and Transformers tend to diverge noticeably because of the fundamentally different modeling paradigms. DALF enables precise alignment and refined fusion of low-level features from different branches, which preserves spatial details and boundary integrity. As shallow CNN and Transformer features often differ in local structure, we introduce a dynamic attention mechanism to explicitly calibrate cross-branch correspondence at low levels. The attention mechanism adaptively models the relevance between local regions from both branches and establishes weight distributions. This greatly enhances the responses of discriminative details (e.g., object edges) while suppressing irrelevant background noise.

Fed into DALF, $T_{i}\;and\;C_{i}$ first pass through independent 1 × 1 convolutions. This lightweight projection aligns the channel spaces of the two branches, making subsequent interactions more stable and comparable. Next, we construct an initial fused representation $F_{i}^{init}$, which is formulated as:

(1) $${t_{i}=Conv}_{1\times 1}\left(T_{i}\right),$$

(2) $$c_{i}={Conv}_{1\times 1}(C_{i}),$$

(3) $$F_{i}^{init}=\left(t_{i}⊗c_{i}\right)⊕t_{i}⊕c_{i},$$

where ⊗ means pixel-wise multiplication and ⊕ denotes element-wise addition. ${Conv}_{1\times 1}(\cdot )$ means the 1×1  convolution operation.

DALF generates query ($Q_{i}$), key ($K_{i}$), and value ($V_{i}$) embeddings to model cross-branch local correlation. The fused representation serves as the value to represent the joint context, while branch-specific features provide the key and query to measure and transfer complementary responses. Distinct from standard attention, DALF computes position-specific weight distributions through element-wise multiplication along the channel dimension. This localized recalibration adaptively highlights discriminative structures while suppressing branch-specific noise, which is formulated as:

(4) $$V_{i}={Conv}_{1\times 1}(F_{i}^{init}),$$

(5) $$K_{i}={Conv}_{1\times 1}(t_{i}),$$

(6) $$Q_{i}={Conv}_{1\times 1}(c_{i}),$$

(7) $$atten=Softmax(K_{i}⊗Q_{i},dim=1),$$

where Softmax(·,dim=1) denotes normalizing the channel dimension to obtain the channel attention weights for each spatial position.

The value feature is dynamically weighted by the attention map to highlight correlated channels. The original query and the weighted query are then concatenated along the channel dimension. A final 1 × 1 convolution projects the concatenated feature into the target dimension to generate the fused output $F_{i}^{fuse}$, which can be expressed as:

(8) $${V_{i}}^{w}=V_{i}⊗atten,$$

(9) $$F_{i}^{cat}=Cat(V_{i},{V_{i}}^{w}),$$

(10) $$F_{i}^{fuse}={Conv}_{1\times 1}(F_{i}^{cat}),$$

where Cat(·) denotes the feature concatenation operation.

(2) Lightweight high-level aggregation module

High-level features are characterized by low resolution and high semantic abstraction, whose core value lies in providing global semantic information. Given their limited spatial granularity, adopting heavy or highly non-linear fusion mechanisms causes huge computational cost but little improvement. Therefore, we adopt a lightweight aggregation module that maintains semantic integrity with little computational overhead.

To align their statistical distributions and stabilize subsequent fusion, the input high-level features $T_{i}\;and\;C_{i}$ are first processed by the same transformation block consisting of a 1×1 convolution, Batch Normalization, and a ReLU activation, which can be expressed as:

(11) $$t_{i}=C_{br}\left(T_{i}\right),$$

(12) $$c_{i}=C_{br}\;(C_{i}),$$

where $C_{br}(\cdot )$ denotes ${Conv}_{1\times 1}+BN+ReLU$ operations. Using the same block encourages the two branches to be mapped into a comparable feature space, which facilitates stable high-level fusion.

(13) $$F_{i}^{init}=t_{i}⊗c_{i}.$$

This multiplicative interaction acts as a semantic gating mechanism, activating consistent high-level responses while suppressing irrelevant features. Next, a residual enhancement strategy is applied, which is formulated as:

(14) $$F_{i}^{fuse}=\;{Conv}_{1\times 1}(F_{i}^{init}⊕c_{i}⊕t_{i}).$$

This residual operation preserves unique semantic cues from each branch and ensures effective high-level semantic transmission.

Through the overall LFI module, the network produces two low-level fused features ($F_{1}^{fuse}$, $F_{2}^{fuse}$) via DALF and two high-level features ($F_{3}^{fuse}$, $F_{4}^{fuse}$) via LHA, which are forwarded to the subsequent cross-attention decoding stages.

### 3.3. Semantic-Aware Edge Refinement Module

Accurate edge cues play a crucial role in saliency prediction, yet many SOD models primarily focus on interior semantics but overlook structural consistency around object contours. Two main challenges restrict existing edge-aware methods: scale mismatch between edge features and semantic features, and lack of interaction between high-level semantic edges and low-level detail edges. To address these issues, we propose SAER, which is composed of MFP and edge calibration enhancement (ECE) submodules. MFP provides lightweight multi-scale edge responses with diverse receptive fields, while ECE injects high-level semantic priors to suppress irrelevant texture details and preserve object-aware contours, effectively implementing the principle of nonequal importance by prioritizing true boundaries.

**Figure 3:**
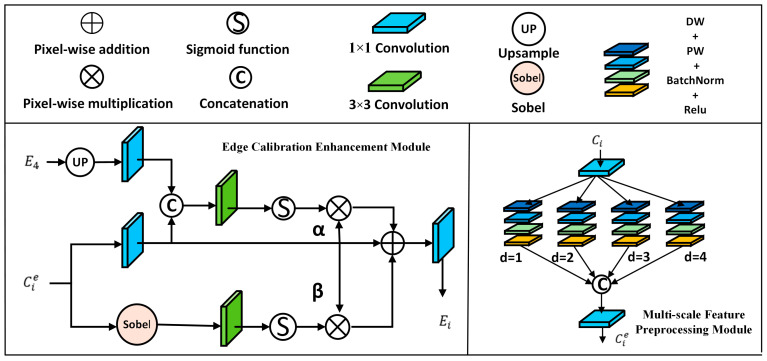
Architecture of the semantic-aware edge refinement module. The SAER consists of an edge calibration enhancement module and a multi-scale feature preprocessing module.

The CNN features enter the MFP module and become $C_{i}^{e}\;\left\{i=1,2,3,4\right\}$. The top-level $C_{4}^{e}$ undergoes an atrous spatial pyramid pooling (ASPP) block and guides other levels in the ECE to calibrate lower-level edge features and yield final multi-scale edge maps $E_{i}\;\left\{i=1,2,3\right\}$. At the same time, edge supervision is applied to each $E_{i}$. An illustration of the module is provided in Figure 3.

(1) Multi-scale feature preprocessing module

The MFP module is designed to extract discriminative edge cues that are robust to object scale variation without introducing heavy computation. Instead of relying on a single receptive field, MFP aggregates parallel dilated depthwise–pointwise branches $\left(d\in \{1,2,3,4\}\right)$, enabling the model to capture both fine contours and broader structural transitions. This design offers a favorable accuracy–efficiency trade-off: depthwise separable convolution operations keep the module lightweight, while multi-dilation aggregation improves edge completeness across diverse object sizes. These operations are formulated as:

(15) $${F_{i\;}}^{\prime }={Conv}_{1\times 1}\left(F_{i\;}\right),$$

(16) $${f_{i}}^{d}=ReLU(PW(DW({F_{i\;}}^{\prime };dilation=d)))\;\left\{d=1,2,3,4\right\},$$

where DW(·) denotes 3 × 3 Depthwise Separable Convolution; PW(·) denotes Pointwise Convolution; BN(·) denotes BatchNorm; ReLU(·) denotes Relu operation.

The outputs are concatenated and fused to produce the multi-scale edge feature:

(17) $$C_{i}^{e}={Conv}_{1\times 1}(Cat({f_{i}}^{1},{f_{i}}^{2},{f_{i}}^{3}{{,f}_{i}}^{4})),$$

where Cat(·) denotes the feature concatenation operation.

(2) Edge calibration enhancement module

ECE introduces the top-level prior $E_{4}$ as semantic guidance to calibrate lower-level edge features $C_{i}^{e}\;\left\{i=1,2,3\right\}$. $E_{4}$ captures global contextual and semantic cues with the largest receptive field, making it well suited as semantic guidance. It is upsampled and compressed to serve as a semantic prior p. Each preliminary edge feature $C_{i}^{e}\;\left\{i=1,2,3\right\}$ is processed in a similar manner, which is formulated as:

(18) $$p={Conv}_{1\times 1}\left(Up\left(E_{4}\right)\right),$$

(19) $$x_{i}={Conv}_{1\times 1}(C_{i}^{e}),$$

where Up(·) denotes upsampling operation.

Specifically, a learned weight $W_{i}$ performs soft semantic selection, encouraging edges consistent with object semantics and suppressing spurious texture boundaries. To complement semantic cues with local gradient signals, we extract an additional attention weight from Sobel-filtered edges. Both semantic and gradient cues are used to refine the edge representation, and we leverage α and β to perform the weighted balance, which is formulated as:

(20) $$W_{i}=\sigma ({Conv}_{3\times 3,padding=1}(Cat(x_{i},p))),$$

(21) $${W_{i}}^{s}=\sigma ({Conv}_{3\times 3,padding=1}(Sobel(C_{i}^{e}))),$$

(22) $$E_{i}=x_{i}⊗(1+\alpha W_{i}+\beta {W_{i}}^{s}),i=1,2,3,$$

where ${Conv}_{3\times 3,padding=1}(\cdot )$ denotes a 3 × 3 convolution with padding = 1; σ(·) denotes the sigmoid activation function. Sobel(·) denotes the Sobel Kernel Operator. The top-level edge feature $E_{4}$ is directly obtained through ASPP without further calibration.

Through the cooperation of MFP and ECE, the SAER module produces multi-scale edge features that are both structurally accurate and semantically consistent. These refined edge cues provide strong structural guidance for the subsequent ECFA.

### 3.4. Edge-Guided Cross-Attention Feature Aggregation Module

**Figure 4:**
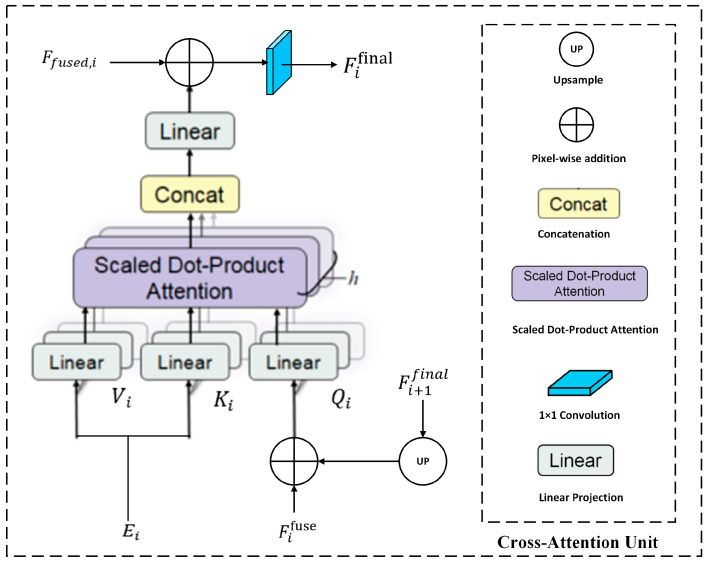
Structure of the ECFA module.

By employing a multi-stage cross-attention mechanism, ECFA explicitly models the interaction between refined edge features $E_{i}$ and semantic features $F_{i}^{fuse}$, enabling a strong structure–semantics synergy and guiding the network to maintain strict spatial continuity within the salient object. ECFA is designed following a top-down progressive refinement cascaded logic, in which high-level semantics guide the reconstruction of fine boundary structures at lower levels. The overall process is divided into four stages (corresponding to the four-scale features of the main branch), as shown in Figure 4. The output of the previous stage serves as a “guidance signal” for the next stage, forming an iterative optimization process.

At the i-th stage, the semantic query feature is generated by combining the fused feature $F_{i}^{fuse}$ with the upsampled output of the previous stage. Let $F_{i}^{final}$ denote the output from stage i+1, initialized to zero when i=4. The query and the key–value embeddings are computed as:

(23) $${F_{i}^{q}=Conv}_{1\times 1}(F_{i}^{fuse}+Up(F_{i+1}^{final})),$$

(24) $$E_{i}^{k}{=Conv}_{1\times 1}\left(E_{i}\right),$$

(25) $${E_{i}^{v}=Conv}_{1\times 1}\left(E_{i}\right),$$

where ${Conv}_{1\times 1}(\cdot )$ denotes a linear projection.

We then perform multi-head cross-attention to model structure–semantics correspondence. Specifically, $F_{i}^{q}$, $E_{i}^{k}$, and $E_{i}^{v}$ are split into h groups along the channel dimension. For the j−th head, scaled dot-product attention is computed as:

(26) $$A_{j}=Softmax\left(\frac{F_{i}^{q}{E_{i}^{k}}^{T}}{\sqrt{D}}\right),$$

(27) $$Y_{i}=A_{j}E_{i}^{v},\;j=1,\ldots ,h,$$

where $\sqrt{D}$ is a scaling factor used to alleviate the gradient vanishing problem of Softmax caused by excessively large attention scores; *h* is the number of attention heads; *T* is the matrix transpose operation.

The head outputs are concatenated and compressed to obtain the attention-enhanced feature. Finally, ECFA merges the structure-aware attention result with same-scale semantics via concatenation and projection, which can be expressed as:

(28) $$Y_{i}={Conv}_{1\times 1}\left(Cat\left(Y_{1}+Y_{2}+Y_{3}+\cdots \ldots Y_{h}\right)\right),$$

(29) $$F_{i}^{final}={Conv}_{1\times 1}(Cat(Y_{i},F_{i}^{fuse})).$$

The fused feature $F_{i}^{final}$ is then upsampled and passed to the next lower stage to guide subsequent refinement. After four cascaded stages, the final output $F_{i}^{final}$ is used to generate the saliency prediction results.

### 3.5. Loss Function

To jointly optimize salient region localization and boundary accuracy, our network adopts a multi-task loss that supervises both the saliency prediction and the multi-scale edge maps generated by the edge branch.

(1) Saliency Supervision

Given the predicted saliency logits S and the ground-truth saliency map $G_{s}$, we employ a standard pixel-wise binary cross-entropy (BCE) loss:

The BCE loss is defined as:

(30) $$L_{BCE}\left(G_{s},P\right)=-\sum_{i=1}^{H}\sum_{j=1}^{W}\left[G_{s}\left(i,j\right)\log\left(P\left(i,j\right)\right)+\left(1-G_{s}\left(i,j\right)\right)\log\left(1-P\left(i,j\right)\right)\right],$$

where H and W denote the height and width of the image; $\left(i,j\right)$ denotes pixel coordinate; $P\left(i,j\right)$ denotes predicted saliency probability; $G_{s}\left(i,j\right)$ denotes saliency ground truth label.

The Dice loss is formulated as:

(31) $$L_{Dice\;}\left(G_{s},P\right)=1-\frac{2\sum_{i,j}P\left(i,j\right)G_{s}\left(i,j\right)}{\sum_{i,j}P\left(i,j\right)+\sum_{i,j}G_{s}\left(i,j\right)}.$$

Thus, the saliency loss is:

(32) $$L_{\mathrm{sal}}=L_{BCE}\left(G_{s},P\right)+L_{Dice\;}\left(G_{s},P\right).$$

(2) Edge Supervision

Given the predicted edge map B and its binary ground truth $G_{e}$, the BCE loss is defined as:

(33) $$L_{BCE}\left(G_{e},B\right)=-\sum_{i=1}^{H}\sum_{j=1}^{W}\left[G_{e}\left(i,j\right)\log\left(B\left(i,j\right)\right)+\left(1-G_{e}\left(i,j\right)\right)\log\left(1-B\left(i,j\right)\right)\right].$$

The Dice loss is formulated as:

(34) $$L_{Dice\;}\left(G_{e},B\right)=1-\frac{2\sum_{i,j}B\left(i,j\right)G_{e}\left(i,j\right)}{\sum_{i,j}B\left(i,j\right)+\sum_{i,j}G_{e}\left(i,j\right)}.$$

Thus, the edge loss is:

(35) $$L_{\mathrm{edge}}=L_{BCE}\left(G_{e},B\right)+L_{Dice\;}\left(G_{e},B\right).$$

(3) Final loss

(36) $$L_{\mathrm{total}}=L_{\mathrm{sal}}+{{ℷ}_{e}L}_{\mathrm{edge}},$$

where ${ℷ}_{e}$ is the edge-loss weighting factor, balancing the relative importance of edge refinement. A larger ${ℷ}_{e}$ encourages sharper boundaries, whereas a smaller value emphasizes region-level accuracy.

## 4. Experimental Results and Analysis

### 4.1. Datasets

We conduct experiments on five widely used SOD benchmark datasets, including DUTS [29], ECSSD [30], HKU-IS [31], DUT-OMRON [32] and PASCAL-S [33] datasets. These benchmarks consist of visual signals captured by various image sensors across diverse scenes and lighting conditions. This inherent diversity ensures that the trained models are robust and adaptive to various acquisition settings, rather than being restricted to specific hardware or fixed illumination. DUTS is currently the largest SOD dataset, which contains 10,553 training images (DUTS-TR) and 5019 testing images (DUTS-TE) collected from various real-world scenes with diverse salient objects and complex backgrounds. DUT-OMRON consists of 5168 high-resolution images with multiple objects and cluttered backgrounds, including eye-fixation records captured via a Tobii X1 Light Eye Tracker (Tobii AB, Danderyd, Sweden) sensor to provide biological ground-truth for evaluating visual saliency. HKU-IS includes 4447 images, most of which contain multiple disconnected salient regions or low-contrast foregrounds. ECSSD is composed of 1000 structurally complex images with rich semantic content, while PASCAL-S provides 850 images with complicated object–background interactions and human gaze data recorded using an EyeLink 1000 tracker (SR Research Ltd., Kanata, ON, Canada) sensor. For preprocessing, all inputs were resized to 320×320 pixels via bilinear interpolation and normalized using standard channel-wise mean and standard deviation. We use DUTS-TR to train our model, and the remaining datasets are used to test.

### 4.2. Evaluation Metrics

To conduct a comprehensive assessment of our model, we report precision-recall (PR) curves and F-measure curves, as well as four scalar metrics: F-measure $\left(F_{\beta }\right)$ [34], Mean Absolute Error (MAE) [35], Structure measure (S) [36] and Boundary Displacement Error (BDE) [37].

(1) F-measure $\left(F_{\beta }\right)$

To jointly consider both precision and recall, we adopt the weighted harmonic mean max-F. Following common practice, we use an adaptive threshold to binarize the predicted saliency map:

(37) $$\begin{array}{c} F_{\beta }=\frac{\left(1+\beta^{2}\right)\times \;\mathrm{Precision}\;\times \;\mathrm{Recall}\;}{\beta^{2}\times \left(\;\mathrm{Precision}\;+\;\mathrm{Recall}\;\right)}, \end{array}$$

where *β* is empirically set to 0.3 to assign more importance to precision than recall in saliency evaluation.

(2) Mean Absolute Error (*MAE*)

MAE measures the average pixel-wise absolute error between the predicted saliency map S and the ground truth G, which is defined as:

(38) $$\begin{array}{c} MAE=\frac{1}{H\times W}\sum_{i=1}^{H}\sum_{j=1}^{W}\left|S_{ij}-G_{ij}\right|. \end{array}$$

(3) Structure Measure *S*

S−measure evaluates structural similarity by jointly modeling object-aware similarity ($S_{o}$) and region-aware similarity ($S_{r}$). It aligns well with human perception of structural consistency, which is represented as:

(39) $$\begin{array}{c} S=\beta S_{o}+\left(1-\beta \right)S_{r}, \end{array}$$

where β balances object-level and region-level components.

(4) Boundary Displacement Error (BDE)

*BDE* is employed to evaluate the accuracy of boundary localization in salient object detection:

(40) $$\begin{array}{c} BDE(X,Y)=\frac{1}{2}[\underset{x}{\sum }\underset{y\in Y}{min}d(x,y)/N_{X}+\underset{y}{\sum }\underset{x\in X}{min}d(x,y)/N_{Y}] \end{array}$$

where X and Y denote predicted and ground-truth boundary points, $N_{X}$ and $N_{Y}$ are the numbers of points in X and Y.

### 4.3. Experimental Settings

The proposed model is trained on the DUTS-TR dataset [29] using a single NVIDIA GeForce RTX 4060 GPU. The backbone parameters are initialized with ResNet-50 [38] and a Masked Autoencoder (MAE) pretrained ViT-Large model [39]. We follow common practice in large-scale Transformer training to freeze the first 20 layers of the MAE-based ViT-Large encoder, aiming to stabilize optimization and reduce memory consumption.

We resize all training images and ground-truth masks to 320×320, as we did for the testing images. SECA-Net is trained for 30 epochs with an initial learning rate of 0.0001, which is decayed to 0.00001 after 15 epochs. The batch size is set to 1, while gradient accumulation is set to 4 to ensure stable optimization of the Transformer branch under limited GPU memory. In addition, we set the edge-loss weight ${ℷ}_{e}$ to 0.25 by default.

The final model contains 327.29 M parameters in total, among which 77.69 M parameters are trainable due to the partial freezing of the ViT-Large encoder. In terms of complexity, the additional computation mainly comes from the dual-branch encoder and the ECFA decoder, while LFI and SAER are implemented with lightweight 1×1 projections and depthwise–pointwise convolutions. At test time, the model runs in a single forward pass without multi-scale inference or post-processing, and auxiliary edge supervision is only used during training.

### 4.4. Performance Comparison

We compare our proposed model with 18 state-of-the-art SOD methods, which can be categorized into four groups based on their characteristics: (1) CNN-based methods, including MINet [40], GCPANet [41], DCNet [42], PurNet [43], EDN [44], PDRNet [45], RMCDF [46], TCRNet [47], ICON [48], MENet [49], HCCNet [20]; (2) Transformer-based methods, including VST [50]; (3) Hybrid CNN-Transformer methods, including CTIFNet [19], DSRNet [11], and CAM-Net [28]; (4) Edge-guided methods, including LDF [51], ELSANet [23], DSRNet [11], RECENet [25], DC-Net [52]. Most of them provide public results or release code for reproducible evaluation. The saliency maps are obtained either from the authors or by running the released codes.

(1) Quantitative Comparison

**Figure 5:**
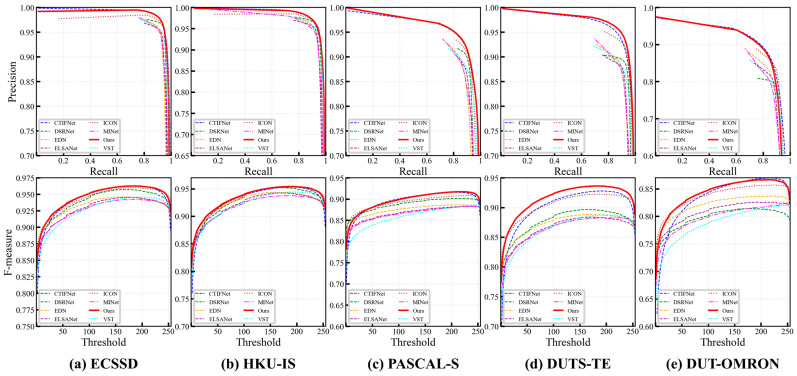
F-measure curves and PR curves of the proposed method and 7 state-of-the-art methods on five datasets.

The quantitative results are shown in Table 1, including $F_{\beta }$, MAE, and S. Our method achieves the best overall performance, especially ranking first in $F_{\beta }$ across all datasets. It also obtains outstanding performance in MAE and S. Particularly, our method improves $F_{\beta }$ by 1.4% on the challenging DUTS-TE dataset, highlighting its superior detection capability. Compared to single-backbone methods, SECA-Net fully leverages the advantages of global modeling and local perception. Specifically, compared with recent CNN-based methods, HCCNet, our model, increases by approximately 1.4% at most on S in DUTS-TE, ECSSD, and PASCAL-S datasets, and performs similarly on the other two datasets. It demonstrates the strong ability of global modeling in complex scenes. Moreover, our method leads Transformer-based methods VST in all aspects of all metrics. Furthermore, the recent Hybrid CNN-Transformer method CTIFNet, which relies on iterative simple feature fusion, achieves better $F_{\beta }$ than CNN-based methods and Transformer-based methods of the same period. Our proposed method introduces SAER and ECFA modules to establish a bidirectional semantic-structure collaboration, showing superior performance. We increased by approximately 1.65% on $F_{\beta }$. Figure 5 shows the $F_{\beta }$ and PR curves across five benchmark datasets with seven state-of-the-art methods. SECA-Net achieves superior performance in all cases, showing stronger threshold robustness and maintaining higher precision across nearly the entire recall range. These results verify that the proposed modules enhance structural fidelity and global consistency, enabling SECA-Net to outperform existing SOTA methods.

We also compare the boundary displacement error (BDE) of the methods selected for $F_{\beta }$ and PR curves in Table 2 to further evaluate the edge learning ability. It reports the BDE results of all competing methods on five benchmark datasets. The proposed SECA-Net achieves the best boundary localization performance on all datasets, demonstrating its strong capability in recovering accurate object contours. Specifically, compared to ELSANet [23], a representative edge-guided method, SECA-Net achieves significantly lower. BDE. While ELSANet attempts to enhance boundary details using local convolutional constraints, it is easily misled by noise inherent in the features extracted from complex backgrounds. In contrast, our SAER module actively filters out irrelevant texture edges under high-level semantic guidance, rather than introducing unpurified edge cues into saliency features at an early stage. These significant improvements highlight the effectiveness of our semantic-aware edge refinement and the edge-guided cross-attention mechanism in handling complex backgrounds and ambiguous boundaries.

In Table 3, we compare our SECA-Net with other state-of-the-art methods in terms of computational complexity, specifically floating-point operations (FLOPs) and the number of parameters (Params). The results are collected from their published papers or calculated based on their officially released codes at their respective testing input sizes. Although freezing the first 20 layers of the ViT encoder reduces the trainable parameters to 77.69 M during training, our dual-backbone architecture inherently leverages the extensive representation power of a large-scale pre-trained model, resulting in a total parameter size of 327.29 M. To fairly evaluate the model scale, we compare SECA-Net with CTIFNet [19], which shares the same ResNet-50 and ViT configuration. As demonstrated in previous quantitative results, our method consistently outperforms CTIFNet across all metrics. Furthermore, despite the large parameter scale, the computational complexity of our model remains highly competitive, requiring only 64.65 GFLOPs.

(2) Qualitative Comparison

**Figure 6:**
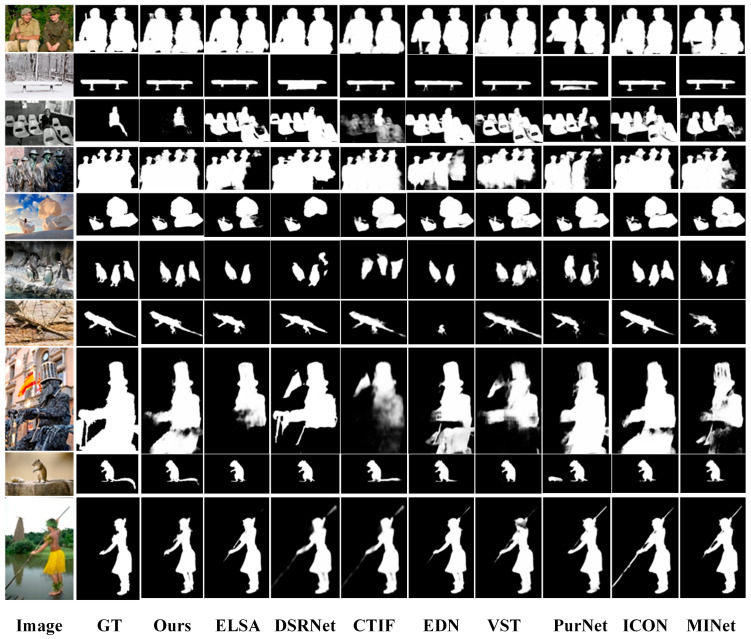
Qualitative comparisons with state-of-the-art methods.

For a more intuitive assessment of SECA-Net, we show visual comparisons over six challenging scene types in Figure 6.

In multiple-object scenarios (Rows 1, 3 and 4), our method clearly separates adjacent subjects and preserves complete object regions. For thin or fine-structured objects (Rows 2, 7 and 10), other methods fail to preserve these fragile structures, whereas SECA-Net captures them intact through SAER. In scenes with small or low-contrast objects (Rows 5 and 9), other methods may overlook salient targets, but SECA-Net accurately detects them with stable and consistent foreground activation. For complex shapes or densely structured objects (Row 8), our method preserves sharp edges and detailed geometry instead of generating incomplete or blurred contours. In texture-similar and visually confusing backgrounds (Row 6), we effectively suppress interference and restore clean object boundaries. In scenarios with foreground disturbance or reflection interference (Row 10), such as water-surface reflections, our method reliably highlights the true salient region while avoiding false activations.

### 4.5. Ablation Studies

To validate the effectiveness of each proposed module, we conduct a series of ablation experiments, which consist of several model variants. All following experiments share the same backbone settings and training strategy. The results on five mainstream datasets are reported in Table 4.

(1) The effectiveness of the ECFA module

To evaluate the contribution of the proposed ECFA module, we replace ECFA with a simple top-down decoder. The absence of cross-level semantic–structure interaction results in noticeably degraded performance, especially in boundary precision and structural consistency. Without this guidance, the network struggles to confine semantic activations within precise contours, leading to blurred boundaries that disrupt the strict spatial continuity of the salient objects.

(2) The effectiveness of the SAER module

To investigate the importance of the edge branch and our core “purify-and-guide” strategy, we conduct two experiments. First, we completely remove the SAER module to investigate the importance of the edge branch. Only the fused features generated by LFI are fed into a top-down decoder. Its performance is significantly weaker compared with the SECA-Net. Second, we only remove the ECE submodule to further explore the importance of purification. In this variant, edge features are extracted solely by the MFP without high-level semantic calibration. As shown in Table 3, the noticeable performance degradation in w/o ECE empirically validates our claim that merely extracting raw edges inevitably introduces texture-induced noise, which is a common flaw in conventional edge-guided methods.

(3) The effectiveness of the LFI module

To analyze the contribution of the LFI module in bridging the semantic gap between CNN and Transformer features, we replace LFI with a simple concatenation followed by convolution. The generated saliency maps exhibit incomplete structural details and reduced global consistency without dynamic interaction and level-specific fusion. Furthermore, to validate the necessity of applying different fusion strategies across different levels, we introduce two variants: Full DALF (applying the DALF module to all levels) and Full LHA (applying the LHA module to all levels). As shown in Table 4, neither variant achieves optimal results. Full DALF introduces unnecessary computational complexity at high levels, where features are already abstract and low-resolution, leading to semantic redundancy and a distortion of global context. Conversely, Full LHA fails at low levels because its lightweight gating mechanism is insufficient to calibrate the spatial misalignment between the CNN’s local receptive fields and the Transformer’s global tokens. The results verify that the proposed multi-level interaction mechanism is more effective than naive fusion strategies.

**Figure 7:**
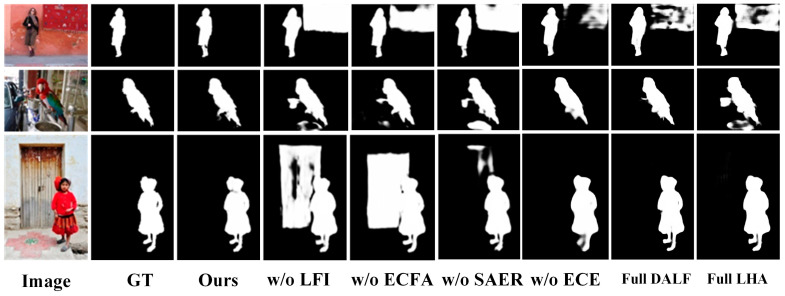
Visual comparisons for showing the benefits of different components.

Additionally, we display some visual comparisons. As shown in Figure 7, the absence of LFI leads to severe background interference, while the exclusion of ECFA causes boundary leakage. Without SAER, the predicted edges become fragmented and structurally inconsistent. In contrast, the proposed full model SECA-Net preserves both global structure and fine-grained boundaries, demonstrating the complementary effects of the designed modules.

### 4.6. Failure Case Analysis

**Figure 8:**
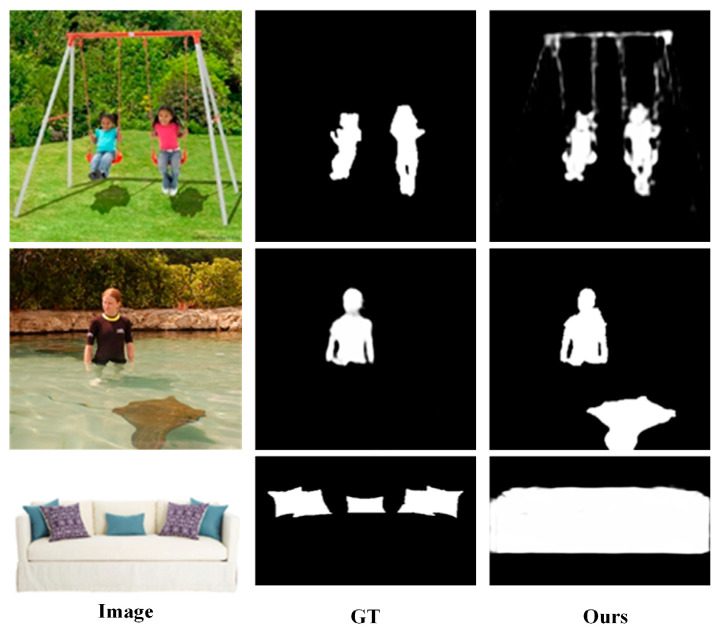
Some failure examples of our proposed method.

While SECA-Net achieves consistent improvements across all benchmark datasets, it nevertheless exhibits limitations in a few extreme scenarios, as illustrated in Figure 8. In scenes containing large homogeneous regions (Row 3), our model detects the entire sofa instead of the pillows. This occurs because the texture-poor background provides limited structural cues, which results in slightly blurred contours. The CNN branch struggles to extract discriminative local structural cues from the texture-poor surfaces, resulting in global information from the Transformer encoder dominating in LFI. In scenes with multiple interacting objects and complex shadows (Row 1), the model focuses on highly contextual components, such as swing ropes. These elements share similar structural patterns with the salient objects, which is harmful to the cross-attention mechanism. Because SAER utilizes high-level semantic guidance to purify edge priors, the rope, which shares structural continuity with the salient region, is mistakenly preserved. As a result, SAER regards the rope as edge cues, thereby interfering with the structure–semantics collaboration in ECFA. In scenarios with submerged underwater objects (Row 2), the model may incorrectly assign saliency to the underwater object rather than focusing solely on the true foreground target. At the feature extraction stage, underwater distractors lead to semantic ambiguity, preventing the encoder from deriving discriminative representations. Consequently, the integrated features generated by LFI contain overlapping semantic responses for both the target and the disturber. This prevents ECFA from accurately suppressing the underwater regions during the final semantic-structure collaboration process.

These failure cases suggest that future work could focus on strengthening the model’s ability to distinguish true object boundaries from context-induced or reflection-induced structures. We could try to introduce stronger semantic–structure disentanglement mechanisms.

## 5. Conclusions

In this paper, we propose SECA-Net, a dual-encoder CNN-Transformer framework that jointly uses global semantic cues and structural information for salient object detection. We design LFI to alleviate the semantic gap between the two heterogeneous encoders. To address the common challenge of edge feature degradation during feature interaction, we further introduce the SAER module to enhance edge representations. ECFA efficiently integrates interior semantics and boundary structures with cross-attention. Extensive experiments on five benchmark datasets display that the proposed SECA-Net achieves outstanding performance, particularly excelling in boundary localization and structural fidelity. In the future, we will investigate more effective utilization of semantic and structural information, achieving a multimodal salient object detection framework of superior performance.

## Figures and Tables

**Table 1:** Results of our model compared with 19 mainstream methods. The best three results are marked in red, green, and blue, respectively. The symbol “↑” indicates a higher value is better, and “↓” indicates a lower value is better.

Methods	Publish	DUTS-TE			ECSSD			HKU-IS			DUT-OMRON			PASCAL-S		
		$F_{\beta }↑$	MAE↓	S↑	$F_{\beta }↑$	MAE↓	S↑	$F_{\beta }↑$	MAE↓	S↑	$F_{\beta }↑$	MAE↓	S↑	$F_{\beta }↑$	MAE↓	S↑
LDF [51]	CVPR20	0.855	0.034	0.892	0.930	0.034	0.925	0.914	0.027	0.920	0.773	0.051	0.839	0.848	0.060	0.861
MINet [40]	CVPR20	0.880	0.037	0.887	0.943	0.036	0.919	0.934	0.029	0.919	0.795	0.056	0.833	0.865	0.064	0.856
GCPANet [41]	AAAI20	0.881	0.038	0.884	0.943	0.037	0.921	0.935	0.032	0.920	0.796	0.057	0.838	0.865	0.063	0.864
VST [50]	ICCV21	0.878	0.037	0.896	0.944	0.033	0.932	0.937	0.029	0.928	0.800	0.058	0.850	0.876	0.060	0.873
DCNet [42]	TIP21	0.877	0.034	0.892	0.938	0.034	0.925	0.929	0.028	0.920	0.782	0.052	0.839	0.855	0.060	0.863
PurNet [43]	TIP21	0.859	0.039	0.869	0.936	0.035	0.925	0.926	0.031	0.918	0.782	0.051	0.841	0.842	0.070	0.850
EDN [44]	TIP22	0.892	0.035	0.895	0.945	0.036	0.919	0.940	0.027	0.922	0.821	0.050	0.849	0.860	0.062	0.865
PDRNet [45]	TCSVT22	0.876	0.035	0.877	0.941	0.032	0.927	0.933	0.027	0.924	0.795	0.052	0.846	0.858	0.061	0.865
RMCDF [46]	RP22	0.855	0.049	0.852	0.934	0.045	0.901	0.929	0.033	0.901	0.817	0.053	0.831	0.853	0.077	0.836
TCRNet [47]	TCSVT23	0.878	0.034	0.880	0.943	0.031	0.928	0.933	0.026	0.923	0.791	0.054	0.843	0.862	0.059	0.865
MENet [49]	CVPR23	0.907	**0.028**	0.908	0.945	0.035	0.918	**0.947**	0.023	0.927	0.812	0.045	0.849	**0.887**	**0.053**	0.872
ICON [48]	TPAMI23	0.891	0.037	0.892	0.946	0.035	0.922	0.939	0.029	0.920	0.821	0.057	0.844	0.876	0.064	0.861
ELSANet [23]	TCSVT24	0.882	0.034	/	0.943	0.030	/	0.935	0.025	/	0.794	0.050	/	0.862	0.059	/
DSRNet [11]	TCSVT24	0.895	0.029	0.903	**0.953**	**0.023**	**0.936**	0.942	**0.021**	0.929	0.810	0.051	0.852	0.880	**0.049**	**0.875**
CTIFNet [19]	TCSVT24	**0.910**	0.030	**0.914**	**0.954**	0.033	**0.933**	**0.944**	0.029	**0.930**	**0.840**	0.049	**0.864**	**0.899**	**0.052**	**0.892**
RECENet [25]	KSII25	0.854	0.041	/	0.926	0.037	/	0.916	0.031	/	0.780	0.059	/	0.851	0.070	/
DC-Net [52]	PR25	0.899	0.032	0.896	0.949	0.034	0.924	0.942	0.027	0.924	0.827	0.053	0.849	0.874	0.066	0.857
CAM-Net [28]	JEIT25	0.900	**0.024**	/	0.0951	**0.023**	/			/	**0.829**	**0.035**	/			/
HCCNet [20]	TIM25	**0.911**	**0.025**	**0.917**	0.945	**0.029**	0.931	**0.949**	**0.020**	**0.938**	0.827	**0.042**	**0.868**	0.878	**0.053**	**0.880**
Ours	/	**0.925**	**0.024**	**0.92** **6**	**0.959**	**0.027**	**0.9** **41**	**0.94** **9**	**0.025**	**0.9** **36**	**0.844**	**0.046**	**0.8** **63**	**0.907**	**0.049**	**0.892**

**Table 2:** Comparison results of our method with the other seven methods in terms of the BDE metric. The best is highlighted in red, respectively. The symbol “↓” indicates a lower value is better.

Methods	DUTS-TE	ECSSD	HKU-IS	DUT-OMRON	PASCAL-S
	BDE↓	BDE↓	BDE↓	BDE↓	BDE↓
MINet [40]	7.29	4.42	3.98	12.66	11.36
EDN [44]	6.66	4.21	3.70	11.04	11.09
VST [50]	6.26	3.74	3.34	12.24	11.39
CTIFNet [19]	4.77	3.07	3.06	11.59	9.12
DSRNet [11]	4.82	3.11	2.97	10.97	9.95
ELSANet [23]	6.53	4.02	3.63	11.40	11.26
ICON [48]	4.30	2.98	2.83	9.69	9.73
Ours	**3.68**	**2.95**	**2.69**	**9.04**	**8.82**

**Table 3:** Comparison of our method with state-of-the-art networks in FLOPs and parameters.

Methods	Backbones	Input Sizs	FLOPs (G)	Params
VST [50]	T2T-ViT	224×224	23.24	44.09
TCRNet [47]	ResNet50	320×320	20.12	57.82
MINet [40]	VGG-16	320×320	87.03	162.38
ELSANet [23]	ResNet50	320×320	21.77	31.92
ICON [48]	ResNet50	384×384	24.96	33.04
DSRNet [11]	Res2Net + P2T	224×224	19.18	115.84
CTIFNet [19]	ResNet50 + ViT	224×224	65.64	328.12
HCCNet [20]	P2T	/	34.41	94.85
Ours	ResNet50 + ViT	320×320	64.65	327.29

**Table 4:** Quantitative comparisons between our model and different variants. The best is highlighted in red. The symbol “↑” indicates a higher value is better, and “↓” indicates a lower value is better.

Method	DUTS-TE			HKU-IS			DUT-OMRON		
	$F_{\beta }↑$	MAE↓	S↑	$F_{\beta }↑$	MAE↓	S↑	$F_{\beta }↑$	MAE↓	S↑
w/o ECFA	0.895	0.035	0.887	0.941	0.027	0.920	0.823	0.060	0.831
w/o SAER	0.904	0.028	0.902	0.941	0.027	0.924	0.831	0.049	0.847
w/o ECE	0.912	0.028	0.909	0.945	0.026	0.928	0.840	0.052	0.855
w/o LFI	0.907	0.029	0.905	0.944	**0.025**	0.928	0.837	0.051	0.852
Full DALF	0.914	0.028	0.912	0.947	0.026	0.931	0.841	0.050	0.856
Full LHA	0.909	0.030	0.907	0.944	0.026	0.927	0.839	0.053	0.853
Ours	**0.925**	**0.024**	**0.926**	**0.949**	**0.025**	**0.936**	**0.844**	**0.046**	**0.863**

## Data Availability

Data are contained within the article.
